# Multidomain Behavioral Change Digital Coaching for Chronic Disease Management in Patients With Type 2 Diabetes: Framework Development and Preliminary Evaluation

**DOI:** 10.2196/73807

**Published:** 2025-07-09

**Authors:** Konstantina Kostopoulou, Danae Lekka, Aristodemos Pnevmatikakis, Nelina Angelova, Panagiotis Stafylas, Stefanos Tamouridis, Alexandra Bargiota, Sofoklis Kyriazakos

**Affiliations:** 1 Innovation Sprint Srl Brussels Belgium; 2 Healthink Thessaloniki Greece; 3 University of Thessaly Volos Greece

**Keywords:** behavior change, mobile health, mHealth, remote patient monitoring, RPM, digital therapeutics, DTx, software as a medical device, SaMD, Healthentia, artificial intelligence, AI

## Abstract

**Background:**

Unhealthy lifestyle behaviors have been identified as a major cause of numerous health issues, with a steady global increase in their prevalence. Addressing this challenge requires comprehensive behavioral changes to promote the adoption of a sustainable healthier lifestyle. However, despite the prevalent need, cost-effective and successful digital coaching for health-related behavior change remains scarce.

**Objective:**

This study aimed to present a holistic framework for designing, modeling, and executing behavior change strategies through a multiagent reasoning system that selected optimal digital coaching techniques based on individual assessments and integrated data-driven decision-making.

**Methods:**

Behavioral change theories have been explored to design a multiagent system aimed at achieving sustainable lifestyle changes. This system selected behavior change techniques based on individual user assessments, prioritizing those with the strongest impact on key behavioral components. The framework incorporated evidence-based practices stemming from behavioral change science and integrated them into Healthentia’s behavioral change coaching scheme. Healthentia, a certified software as a medical device, implemented this framework in its non-medical modules that aim for lifestyle behavioral change and wellbeing specifically for chronic disease management, serving as an eHealth solution that advances decentralized care by enabling remote monitoring, data-driven content selection, and personalized digital coaching that adjusts to patient progress and engagement patterns.

**Results:**

This study explored the application of the Healthentia behavioral change coaching scheme in patients with type 2 diabetes. Behavioral attributes have been evaluated in 9 patients, yielding notable results in terms of fasting glucose dropping by an average of –17.3 mg/dL (Cohen *d*=1.5; *P*=.002), further underscored by a narrow 95% CI (–26.1 to –8.43), and in terms of weight and BMI, with mean reductions of –2.89 kg and –1.05 kg/m², respectively. These changes yielded large effect sizes (Cohen *d* approximately 1.05) and were statistically significant (*P*=.01). The positive outcomes were at least partly attributed to the personalized delivery of content, 71.66% (1125/1570) of which was well received by the patients.

**Conclusions:**

Our study of this multiagent system, which was tested through simulated patient behavior and preliminary, limited behavior observations of patients with type 2 diabetes, promises improved health outcomes using personalized digital coaching strategies. Future directions include optimizing the multiagent selection process; further exploring the type 2 diabetes program; conducting an in-depth evaluation of its results, including glycated hemoglobin measurements; and expanding its applications to other chronic conditions.

## Introduction

### Background and Significance

The escalating global prevalence of lifestyle diseases, such as obesity, diabetes, and cardiovascular conditions, has become a critical health challenge that demands innovative digital coaching to enable behavioral change. These chronic conditions, exacerbated by sedentary lifestyles and accompanied by poor dietary habits, insufficient physical activity, and unhealthy habits such as smoking, reflect broader societal patterns of behavior that compromise population health and increase premature mortality. These diseases combined are responsible for >70% of global deaths.

Traditional medical approaches focused solely on treatment have proven insufficient in addressing the root causes of these health challenges. A more comprehensive approach is necessary to address the complex interplay between individual behaviors, environmental factors, and systemic health determinants. Healthentia (product of Innovation Sprint) attempts to achieve this by integrating behavioral change digital coaching on top of remote standard-of-care monitoring offered to patients with chronic diseases, presenting a promising approach to targeting the underlying mechanisms that contribute to unhealthy lifestyle choices.

### Technological Approach

Healthentia platform [1-4] is a class IIa–certified software as a medical device intended for (1) the collection and transmission of physiological data, including heart rate, blood pressure, oxygen saturation, and weight, directly to health care providers via automated electronic means, in combination with validated Internet of Things devices; (2) the visualization (patient-based dashboards) and the mathematical treatment of data (trends analysis and alerts) related to the monitored physiological parameters of patients with chronic diseases; (3) the transmission of patient’s outcomes and outcome scores related to patient’s health status, health-affecting factors, health-related quality of life, disease knowledge, and adherence to treatment through validated questionnaires; and (4) user (patient) interaction with a conversational digital coach for informative and motivational purposes, supporting patient telemonitoring, decision-making, and behavioral coaching.

Healthentia has 2 components: a mobile app for patients and a web portal for health care professionals. The platform can be customized for different programs and therapeutic areas with a collection of medical and nonmedical modules. The medical modules of the device are intended to support patient telemonitoring, decision-making, and digital coaching by collecting and transmitting physiological data through validated Internet of Things devices. They also enable data visualization and their mathematical treatment, transmission of patient outcomes through validated questionnaires, and interaction with a conversational digital coach. Further to the remote patient monitoring features of Healthentia, which are part of the medical modules under the CE-mark, other non-medical modules such as the smart services, drive digital therapeutics features that aim to improve patients’ lifestyle and wellbeing using a novel, behavioral change–based digital coaching approach.

### Conceptual Framework

To empower individuals to make sustainable lifestyle changes, we need to understand and modify the cognitive, motivational, and environmental factors that influence health behaviors. To accomplish this, the theoretical frameworks that apply to behavioral change are examined. An essential element of success for lifestyle changes is to achieve patient empowerment by training them in self-management of their condition, enhancing therapy adherence, and improving health outcomes. The use of smartphones, which have become essential daily tools, has emerged as an effective strategy for promoting self-management by providing access to health information and coaching [5]. In recent years, many mobile health apps delivering personalized digital coaching have emerged, offering cost-effective and convenient at-home support; however, most of them lack embedded evidence-based behavior change theories, limiting their impact on long-term, sustainable health outcomes [6].

The proposed framework aims to address multiple gaps within the existing landscape of digital coaching. By implementing multidomain behavioral change agents, the framework seeks to provide comprehensive support tailored to individual patient needs. Many current digital coaching systems lack real-time evaluations and personalized feedback, particularly in home-based settings. Immediate or close to immediate feedback, combined with emotional and psychological support, creates a framework that offers holistic care by addressing various aspects of well-being through agents and their multidimensional parameters. Furthermore, addressing the notable gap in seamless integration of digital coaching systems with established clinical pathways, the framework aligns the digital coaching system with existing clinical protocols, improving patient outcomes by enhancing the continuity of care.

### Objectives and Hypothesis

In this study, we aimed to present a framework for personalized behavior change coaching (BCC), which integrates widely used theoretical models, and describe the implementation of this framework within the Healthentia platform for type 2 diabetes mellitus (T2DM) management. A detailed process of each phase of this framework has been described, establishing its conceptual and operational foundations. In addition, preliminary results from an ongoing pilot study have been shared to assess feasibility, engagement metrics, and initial clinical outcomes. While the preliminary results can provide suggestive trends and early indications, full validation of this framework will require more data collection and analysis. We hypothesized that personalized digital coaching, rooted in behavioral science and aligned with an individual’s unique beliefs, capabilities, and motivations, would demonstrate higher engagement and retention rates, potentially enhancing patient self-management strategies [7].

## Methods

### Overview

Developing, implementing, and evaluating behavior change techniques (BCTs) to change established behaviors can be challenging. A robust theoretical framework is essential for backing up any type of behavior change, as it enables predicting the desired outcomes, guides the digital coaching design by establishing the success criteria, and facilitates empirical evaluation and replication.

According to Michie et al [8] and the UK Medical Research Council, a strategic, systematic approach for designing behavioral change programs consisted of 3 phases, grounded in scientific understanding of behavior change mechanisms and moving from theoretical conceptualization to practical implementation [8]. First was the “theory” phase, where the theoretical basis of the BCC program was developed. The “modeling” phase hypothesized the behavioral determinants and identified possible targets and ways of changing them by testing the BCTs. The final one was the “experimental” phase, during which exploratory trials assessed the findings. This part of the paper analyzed these 3 phases and the way they were incorporated into our overall framework.

### Theory Phase

#### Overview

To design behavioral change–based digital coaching, we looked for theories following a systematic approach, as there is evidence supporting that following theoretical constructs is more effective than relying solely on factors such as age or sex [8]. Although there exist many theories and models for designing behavioral change approaches, there is limited guidance on how to effectively implement them in practice.

Our systematic approach integrated 3 complementary theoretical frameworks. The *Behaviour Change Wheel (BCW)* was used to design the program based on unique behavioral, population, and setting characteristics [9,10], linking evidence-based intervention functions and digital coaching methods to a behavioral model at the core. This core model was the *capability, opportunity, motivation, and behavior (COM-B) model,* the second framework used, which helped us understand behaviors in terms of their determinants, further linking them to BCTs as their implementation strategies. The last framework that was integrated into our approach was the *belief-desire-intention (BDI)* framework, which was used to model the interactions between a coachee’s multiple risk behaviors, a coach’s decision-making process for selecting the most appropriate technique or content to achieve the optimum outcome, and the actual expected effect on the behavior.

All these frameworks had distinct yet complementary roles across the 3 phases introduced in the Overview section. In the theory phase, the COM-B model defined the behavioral determinants that had to be targeted. BCW mapped COM-B components to specific intervention functions, and the BDI framework established the foundation for modeling decision-making processes.

In the modeling phase, the COM-B model was the basis for user assessment, translating raw data into their behavioral constructs. BCW guided the selection and prioritization of BCTs, and finally, BDI provided the computational architecture for simulating different patient behaviors and coaching responses.

In the experimental phase, the COM-B model was again used as the patient assessment framework; however, it was also used to measure the real-time changes in behavioral determinants. BCW was used in a manner similar to the modeling phase, and the BDI framework drove the real-time selection and adaptation of coaching strategies.

More details of the modeling and experimental phases have been provided in the subsequent sections. A detailed explanation of the theoretical frameworks has been presented in the remainder of this section.

#### BCW and the COM-B Model

BCW synthesized 19 different frameworks into a single comprehensive approach of 2 layers: the intervention *function layer* comprised 9 functions (ie, education, persuasion, incentivization, coercion, training, enablement, modeling, environmental restructuring, and restrictions) and the *policy categories layer* comprised 7 categories, linked at their core with the COM-B model.

The COM-B model identified 3 essential components for behavior change: *capability* (physical and psychological), which evaluated the knowledge and skills required and the physical ability to perform; *opportunity* (social and physical), which identified environmental factors and social influences; and *motivation* (reflective and automatic), which reflected the conscious decision-making as well as the unconscious responses and emotional reactions [11]. BCW defined behavior as an interaction between the 3 necessary components of the COM-B model [9].

**Figure 1:**
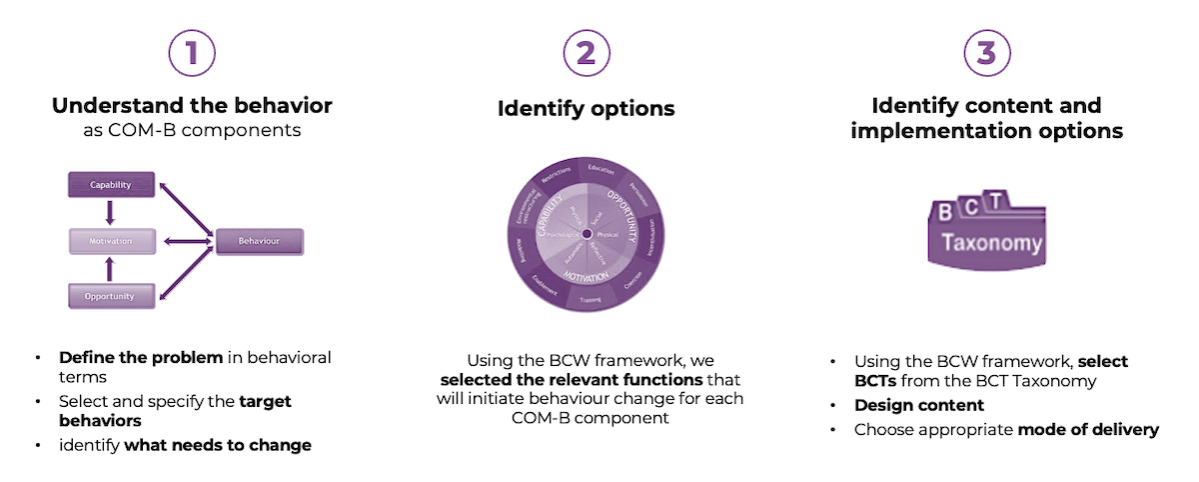
Design process. BCT: behavior change technique; BCW: Behaviour Change Wheel; COM-B: capability, opportunity, motivation, and behavior.

The BCW design process has 3 stages, as shown in Figure 1.

The COM-B components assessed behavior in terms of theoretical behavioral domains, identifying which human behavioral aspects could be influenced to achieve behavior change. These domains could then be targeted through multidimensional processes called “mechanisms of actions” [12], which drove the behavior change and allowed the understanding of “why” certain BCTs changed behaviors, giving us proof of effectiveness. The theoretical domains were addressed by implementing BCTs that addressed the components of ≥1 behaviors separately or simultaneously.

BCTs were identified as standardized digital coaching drawn from a validated taxonomy of 93 identified available techniques [13]. Literature indicated that the combination of multiple BCTs was generally more effective than their single, stand-alone use; therefore, we selected promising combinations based on the findings and tools from the Theory and Techniques of Behavior Change Project [14], effectively linking BCTs with their mechanisms of action.

Literature examples of combining “habit formation” with “self-monitoring” or “environmental restructuring” with “goal setting” showed a particular promise [15,16], as did “self-monitoring” with “goal setting” [17]. However, it is important to mention that the effectiveness of specific BCTs could vary based on the targeted behavior, the individual, and the context. The way and reason behind choosing a technique and the way we monitored their effectiveness to adjust them if needed have been explored further in the *Healthentia BCC* section.

#### BDI Framework

The last challenge was to find a theoretical paradigm to model behavioral change interactions. To determine how a coaching solution could decide which type of digital coaching should be used based on a desired targeted outcome, the theoretical paradigm of the BDI framework was used. The BDI paradigm was developed by Bratman [18] in the 1980s, explaining rational decision-making processes by modeling how agents selected and committed to specific actions based on their understanding of the environment and their desired outcomes.

The BDI framework consisted of three interconnected mental states:

1. Beliefs represented an agent’s knowledge about the world
2. Desires articulated the agent’s goals or objectives
3. Intentions described the planned actions to achieve those desires

This framework provided a structured approach toward understanding how rational agents made decisions, offering insights into goal-directed behavior by demonstrating how intentions emerged from an agent’s existing beliefs and desires.

#### Healthentia BCC

##### Overview

The BCC cycle was the framework that we applied to our solution, integrating the theoretical frameworks mentioned in the Theory Phase section in terms of assessing, modeling, and optimizing behavior change digital coaching. “Digital coaching” is essentially the application of structured BCTs and their digital delivery through the conversational agent of the mobile app, as detailed subsequently.

**Figure 2:**
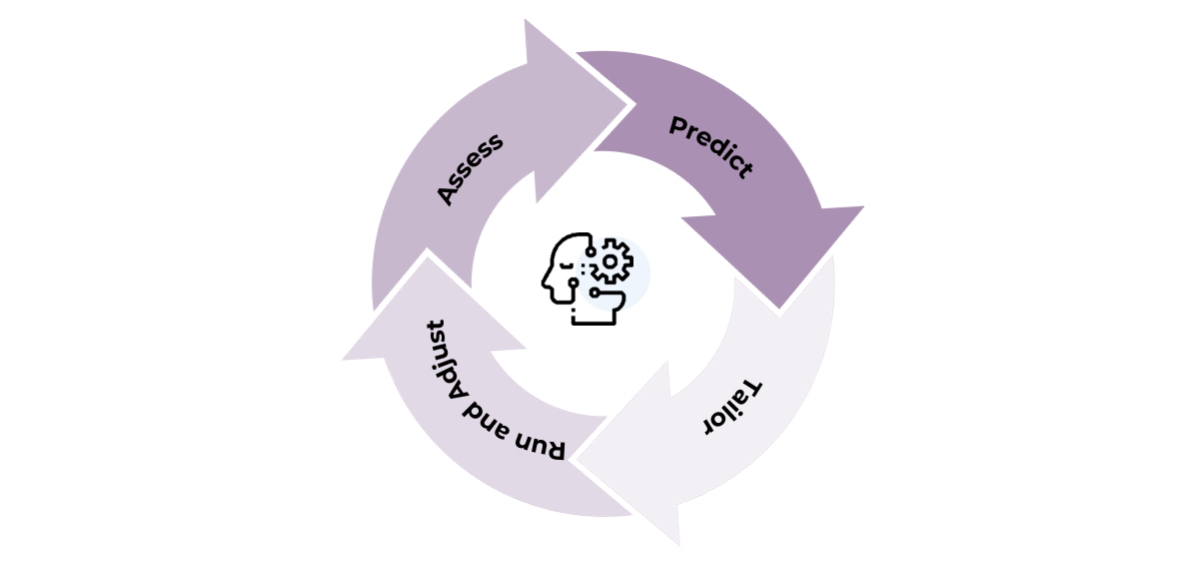
Behavior change coaching cycle.

The framework is depicted in Figure 2 and divided into 4 main steps: assess, predict, tailor, and run and adjust.

##### Step 1: Assess

In this step, reference patient data are collected and calculated to establish a baseline state A, providing insights into their behavior and health status and identifying their key behavioral determinants and characteristics. The data collected in this step might include clinical (health metrics and medical history), demographic (educational, marital, and environmental details), behavioral (baseline activity steps and intensity minutes), and psychosocial indicators (mental state, motivation, social support, and environmental factors).

After data collection, we proceeded to feature engineering, where the collected data are normalized and mapped to COM-B constructs, linking initial behavioral determinants to key domains, such as physical activity, sleep, nutrition, and self-care, and ensuring consistency and relevance for the subsequent modeling phase by effective feature structuring. It focused on the following constructs:

- Capability analysis: evaluating knowledge, skills, and physical attributes
- Opportunity analysis: assessing social and environmental factors
- Motivation analysis: understanding reflective and automatic motivations

##### Step 2: Predict

In this step, the individual feature vector from state A was used, and state B was predicted as the desired outcome after a 12-week digital coaching period.

##### Step 3: Tailor

In this step, based on the 2 states (A and B), a tailored digital coaching process was created, with the selection of domains and specific BCTs based on COM-B scores personalized to the patient.

##### Step 4: Run and Adjust

In this step, the BCTs were applied following the tailored digital coaching plan. The system continuously monitored patient behavior to detect deviations and reassessed the digital coaching plan if deviations exceeded acceptable thresholds.

### Modeling Phase

#### Overview

In the theory phase, the theoretical frameworks of BCW, COM-B, and BDI were introduced, and the integration of these theoretical models into the Healthentia BCC framework was explained. Moving into the modeling phase, the methodology of how we modeled the Healthentia BCC framework and integrated it into our complete product offering has been described as follows.

Healthentia included predefined therapeutic program templates for lifestyle diseases, such as T2DM, heart failure, or chronic obstructive pulmonary disease, and BCC was provided in addition to the capabilities of a standard telemonitoring tool, addressing risk behaviors, such as poor diet, physical inactivity, inadequate sleep, emotional health challenges, and smoking, to improve outcomes. These templates were tailored to the individual needs as part of their personalized program journey.

#### Lifestyle Therapeutic Program Design

In Healthentia, the therapeutic programs designed for lifestyle diseases used a standard-of-care clinical protocol for remote monitoring, which consisted of collecting patient outcomes in the form of surveys and measurements or vital signs at a specified frequency. Each program was orchestrated by an open-source business process modeling and notation tool, which scheduled events and reminders while also streamlining the different phases of the program with all system interactions. The workflows navigated a patient through the requirements of each program phase for a specific timeline, beginning with the assessment phase, where all initial data collection occurred.

For each therapeutic program of a lifestyle disease, we identified the risk behaviors to be targeted using the BCW, as described in the BCW and COM-B Model section. This process helped us address and influence behavior change for all potential needs of patients in the program, by identifying all suitable functions, BCTs, and related content.

#### Behavioral Change Digital Coaching Modeling Design

After having identified the targeted behaviors and BCTs for a specific therapeutic program, a way to initiate the system with a starting plan for real-time execution was needed. In this section, we describe how the BDI paradigm was used to simulate real-world health plans, where digital coaches worked alongside individuals to achieve their goals while considering human factors, such as capability, motivation, fatigue, and personal expectations.

The BCTs were the implementation strategies of digital coaching and could be classified into 3 categories in the system based on their mode of delivery. A BCT could take the form of content (article or media), such as “Instruction on how to perform a behavior.” It could also be a systemic function or feature, such as “Discrepancy between current behavior and goal,” where the system calculated the difference, as well as an interactive dialogue, such as “Problem-Solving,” using the chatting feature.

For the reader’s convenience, a comprehensive glossary of all BCTs mentioned in this paper, including definitions, implementation examples, and mode of delivery, has been provided in Multimedia Appendix 1.

First, a simulating platform was used to model BDI agents per behavioral domain, representing a rational decision-making entity to select the digital coaching and a coachee model to simulate behavioral responses based on the applied plan for different BCTs.

The 3 primary components of each BDI coach agent were as follows:

1. Beliefs:
  inclusion of the current state of the coachees’ behaviors, performance factors, history, and environmental context
2. Desires:
  inclusion of the target behavioral outcomes, optimal COM-B states, and priorities
3. Intentions:
  selection of the appropriate BCTs, constructing a plan, and monitoring it throughout the digital coaching period

The development was initiated by a physical activity coach inspired by Taj et al [19], who simulated a health coaching system that aimed to promote physical activity. Coaching agents for other behavior domains (nutrition, sleep, and self-care) were designed similarly. Some BCTs that we included in this first BDI model were for beliefs (2.1: monitoring of behavior without feedback), desires (1.1 and 1.4: goal setting and planning), and intentions (1.6: discrepancy between current behavior and goal; 2.2: feedback on behavior; 10.3: nonspecific reward; 5.1: information about health consequences, 5.3: information about social and environmental consequences, and 5.6: information about emotional consequences; 6.1: demonstration of the behavior; and 4.1: instruction on how to perform a behavior).

A coachee model represented an individual receiving digital coaching and was modeled based on COM-B constructs, performance factors (eg, fatigue and recovery) across various behavioral domains, and response mechanisms to digital coaching. The modeling of the coachee behavior was inspired by Howlett et al [20], who examined how COM-B components influenced moderate to vigorous physical activity. In this study, COM-B components were mapped to the theoretical behavioral determinants and were measured through validated or custom questionnaires that were benchmarked against a behavioral outcome a week later. In summary, the coachee model depicted how BCTs impact an individual’s COM-B components through direct capability enhancement, opportunity modification, and motivation strengthening.

The effectiveness of digital coaching was assessed and optimized to achieve the predicted behavioral outcome. By testing different combinations of BCTs, the system identified the most effective digital coaching plan for achieving the desired behavior changes. The simulation outputs included predicted outcomes of behaviors across key domains; recommended BCTs and their frequency of use; and generated insights into recovery capacity, resilience, and maintenance potential. These outputs were refined and stored as a starting plan for real-time execution.

#### BCC Initialization

Established models were ready to be fed into the BCC cycle, which provided personalized coaching for enrolled users. After the onboarding of a patient to a program that had behavioral coaching enabled, the process described in the theory phase was initialized by starting with a 2-week long *initialization* phase to collect and process data and assess the patient’s capability, opportunity, and motivation (COM-B) required to build an understanding of the patient’s baseline behaviors. This ensured that digital coaching was tailored to the individual, increasing the likelihood of success.

**Figure 3:**
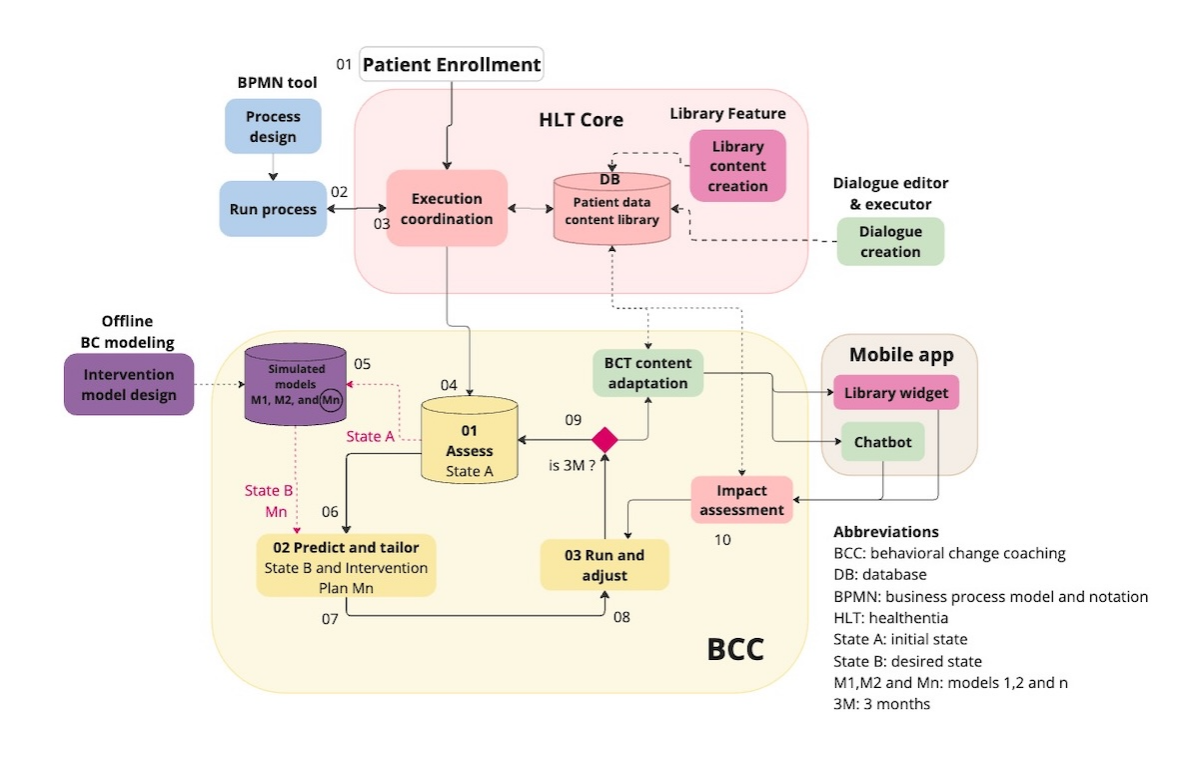
Healthentia behavior change coaching solution.

After the completion of the initialization, the iterative process of the BCC cycle started with steps 1 and 2, and the digital coaching phase kicked off with steps 3 and 4 with personalized coaching. Figure 3 presents the integration of the BCC cycle within the whole product through its connections with other functionalities of the system, such as the mobile app, the dialogue editor, and the executor or the process automation business process modeling and notation tool.

Digital coaching was delivered to the patients through an artificial intelligence–driven conversational agent, “the coach.” Its technical implementation used a rule-based engine embedded within the Healthentia ecosystem and operated autonomously without human intervention, using the BDI framework to select appropriate content and timing based on patient data. The coach could deliver scheduled educational content and respond adaptively to patient-initiated interactions.

It was designed by a multidisciplinary team, including behavioral scientists with expertise in digital health interventions, clinicians specializing in diabetes and cardiovascular disease management, and software engineers.

In practice, the coach is used to deliver the personalized content selected and to engage the users through dialogues. This method provides a more interpersonal and convincing approach to advising users, offers feedback on progress, and addresses barriers or facilitators to help achieve the desired progress toward the targeted behavior. It also allowed for interactive data collection, facilitating continuous adaptation of the digital coaching plan. The frequency and content of interactions between the patient and coach were dynamically adjusted based on each user’s digital coaching plan, needs, and preferences.

The coach was integrated into the mobile app, where users engaged with the delivered content in a chat-based interface. When the BCC system determined that specific content should be shared with the user, it was delivered via the coach as a notification. Users could tap on the notification to expand it, revealing a structured conversational flow that guided them through personalized health information, self-care tips, and digital coaching strategies.

**Figure 4:**
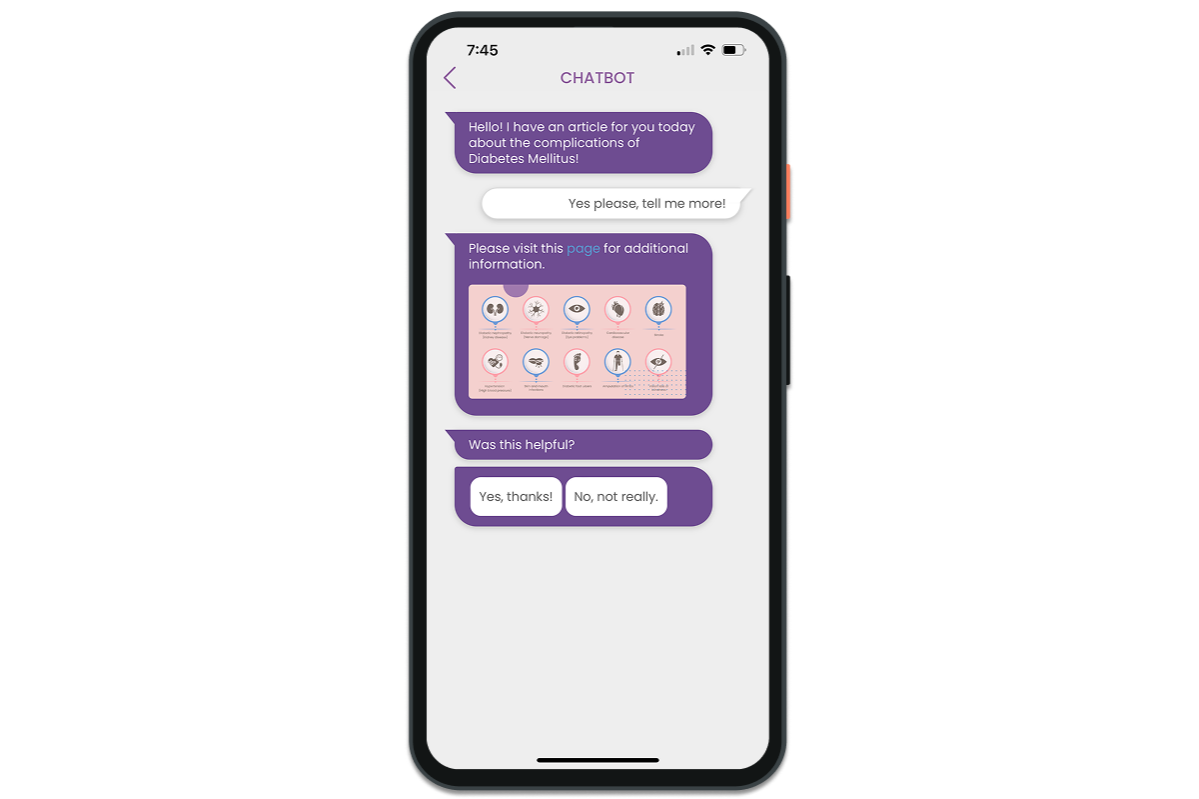
An example of the digital coach in the Healthentia mobile app, assisting a patient with diabetes through an interactive conversation.

Figure 4 shows an example of the conversational agent in the mobile app, where the coach provides information about diabetes mellitus complications. It illustrates the delivery of educational content in an engaging manner, aiming to encourage users to explore further details and assess the content delivered through the mobile app.

### Experimental Phase

#### Study Design and Methods

A program for T2DM was co-designed with clinicians from the General University Hospital of Larissa leveraging BCC principles. This program followed a structured approach integrating behavior change digital coaching with remote clinical monitoring. It aimed to improve self-monitoring, motivation, and adherence, which in turn contributed to improved glycemic control and health outcomes. The objectives of this program were as follows:

- Primary clinical objective: to investigate the effect of the digital coaching on the glycated hemoglobin (HbA1c), as it was measured at the beginning and the end of the program
- Secondary clinical objective: to investigate the effect of digital coaching on blood glucose measurements using factors such as timely digital coaching and acceptance of recommendations
- Behavioral objectives: to investigate the effect of the digital coaching on weight (toward normal BMI range), physical activity (an increase in baseline step count and reaching at least 150 active min spent per week), and sleep (toward a duration between 6 and 8 hour)

Patients with T2DM who met the inclusion criteria and were being treated at the University Clinic of Endocrinology and Metabolic Diseases were recruited to the study. Each enrolled patient participated for a total duration of 14 weeks (2 control weeks of assessment and 12 weeks of behavior change digital coaching). During this period, each patient continued to be treated as usual, receiving standard medical care.

With regard to inclusion criteria, we are currently collecting data from a clinical study involving patients with T2DM, using the inclusion and exclusion criteria outlined in Textbox 1.

**Inclusion and exclusion criteria.**

**Inclusion criteria**

- Glycated hemoglobin value >6.5% and access to a suitable mobile phone (smartphone) device that patients could adequately operate or a caregiver available to enter the patient’s data into the app in case the patient was not familiar with it.
- Receiving one of the following treatments to manage their diabetes:
- Antidiabetic tablets
- Insulin or other injectable treatments
- A combination of the above

**Exclusion criteria**

- Unwillingness to have frequent capillary blood glucose measurements
- Refusal of written informed consent to participate in the study
- Currently using or having received a lifestyle intervention or modification related to diabetes mellitus within the past 12 months

The primary end point was the change in HbA_1c_ levels, while the secondary end points focused on the changes in physical activity, body weight and BMI, sleep habits, smoking cessation, dietary habits, health-related quality of life, and other laboratory parameters.

Following the BCC cycle of Figure 2, the program had an assessment phase and a coaching phase, which integrated the predict, tailor, and run and adjust steps.

The assessment phase had a dual goal:

1. To assess the patient by collecting baseline measurements and initial self-reports via behavioral questionnaires (see full list below, in the last paragraph of this section).
2. To empower the patient by introducing them to Healthentia for their diabetes program. Adherence was tracked by the system and was reported to the health care professional, who decided whether the patient was ready to progress to the behavior change digital coaching phase.

The coaching phase had the following goals:

- Personalized goal setting for steps, intensity minutes, and sleep based on the data collected from the assessment phase and the physician recommendations
- Continuous collection of patient data using a Fitbit tracker (Fitbit, Inc) and manual mobile app inputs
- Acting upon the collected data, the system both tailored the behavioral coaching and delivered it. The BCC system always monitored and evaluated data, prompting reminders and alerts and guiding the coach and its delivery content and timing.

While life-threatening situations were beyond the scope of Healthentia, the diabetes program added to the time-critical alert process for measurements by considering extreme blood glucose values.

Patient data were continuously collected and monitored throughout the BCC-driven diabetes program, including blood glucose measurements, weight measurements, questionnaire responses, and physical activity and sleep data. All data were logged and could be reviewed from Healthentia’s mobile app.

During the first week of assessment, patients were asked to perform structured blood glucose measurements, as indicated by the Healthentia mobile app notifications. This not only provided a baseline for blood glucose levels but also familiarized patients with the app’s notification system and self-reporting process. Throughout the coaching phase, patients continued to track blood glucose levels at predetermined times of the day with greater flexibility, using a pattern designed for patients not meeting glycemic targets, aiming for at least 6 measurements per week. Healthentia’s BCC system monitored adherence, sending reminders when there were too few or not widely spread measurements. It also detected indications of hyperglycemia, encouraging patients to consult their physician if a single extreme value or consistently high measurements were encountered. The structured blood glucose measurements were repeated in the final week of the coaching phase.

Patients were expected to log into the app and enter their weight at least weekly during the coaching phase through a weight widget. Healthentia monitored weight values and sent notifications every Friday to patients who had not logged any values for the current week.

Physical activity and sleep data were collected using a Fitbit activity tracker. During assessment, the average daily steps were collected to establish each patient’s baseline, which was then used to evaluate progress.

The program used 3 custom questionnaires (the COM-B questionnaire; Multimedia Appendix 2 [10]) and 9 validated ones (Fagerstrom Test for Nicotine Dependence, International Physical Activity Questionnaire, Problem Areas in Diabetes, Diabetes Knowledge Questionnaire-24, Diabetes Management Self-Efficacy Scale, EQ-5D, Mediterranean Diet Score, General Anxiety Disorder-7, and Patient Health Questionnaire-9). During the assessment phase, all questionnaires were first sent over the weekend of the first week to be answered during the second week. Reminders were sent periodically for unanswered questionnaires until they expired a week later. During the coaching phase, 3 questionnaires were repeated, offering updated information for the run and adjust step (Mediterranean Diet Score, General Anxiety Disorder-7, and Patient Health Questionnaire-9). During the final week of the coaching phase, all questionnaires were resent, except the Fagerstrom Test, which was replaced by the Poststudy System Usability Questionnaire.

#### Coaching in Action

When the assessment period was completed, all the collected patient data were used to establish a baseline state A, highlighting their key behavioral determinants and characteristics mapped to COM-B constructs. Using expert knowledge and the modeling simulations described in the Methods section, a state B was established and used to prioritize behavioral domain needs by setting personalized goals and selecting the relevant BCTs and content associated with them (eg, tips and lessons).

The “coach,” central to the run and adjust step, sustained patient engagement by delivering personalized content. It delivered three main types of content:

1. From 7 interactive dialogues on goals, 1 was selected and sent every morning to support patients in meeting their goals.
2. From 40 educational tips, 1 was selected and delivered every evening (except Sundays) to both support and educate the patients for improved disease management.
3. From 7 lessons, 1 was selected and sent each Sunday evening, offering more detailed educational material.

The content that could be delivered was dynamically adjusted based on each patient’s needs and preferences. These messages were tailored based on the patient’s progress and BCTs across 4 behavioral domains, including self-care, nutrition, physical activity, and sleep, as shown in Table 1.

**Table 1:** Classification of the content delivered to patients per type, domain, and function. The number of content items is shown in parentheses.

Content type and domain		BCTs^a^
**Lessons (n=7)**		
	Self-care	4.1, 4.2, and 5.1
**Tips (n=40)**		
	Self-care (n=1)	4.1 and 4.2
	Self-care (n=9)	8.2, 7.1, and 15.1
	Nutrition (n=4)	4.1 and 4.2
	Nutrition (n=11)	8.2, 7.1, and 15.1
	Physical activity (n=3)	4.1 and 4.2
	Physical activity (n=3)	8.2, 7.1, and 15.1
	Sleep (n=4)	4.1 and 4.2
	Sleep (n=5)	8.2, 7.1, and 15.1
**Interactive dialogues on goals (n=7)**		
	Self-care (n=2)	1.1-1.7, 2.1, 2.2, 2.6, and 2.7
	Physical activity (n=3)	1.1-1.7, 2.1, 2.2, 2.6, and 2.7
	Sleep (n=2)	1.1-1.7, 2.1, 2.2, 2.6, and 2.7

^a^BCT: behavior change technique.

Examples of some of the BCTs mentioned in Table 1 include the following:

- 4.1: instruction on how to perform a behavior (an article that demonstrated step-by-step how to monitor blood glucose and how to take proper medication [21] and an article that offered tips and guidance for better sleep patterns [22])
- 5.1, 5.3, and 5.6: information about health, social, environmental, and emotional consequences (small text tips were delivered as messages from the digital coach for each of these BCTs)

**Figure 5:**
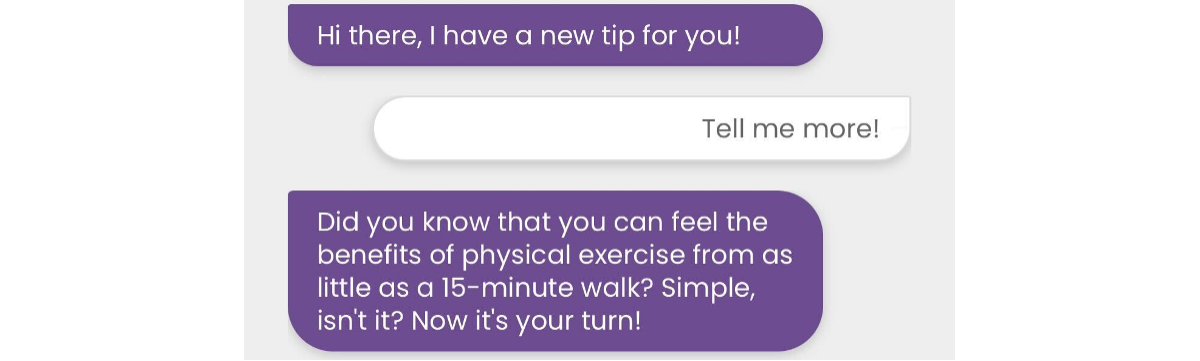
An example of a physical activity tip delivered to a patient with type 2 diabetes.

An example of an evidence-based tip on physical activity is shown in Figure 5. This and most of the physical activity content were based on the study by Colberg et al [23].

The full list of BCTs mentioned in Table 1 is reported in Multimedia Appendix 1. The chatbot interactions were purely text based at this point of the study. However, other media, such as audio or video, were planned to be included as content in the future.

### Ethical Considerations

This open-label observational study, titled “Clinical Intervention Study for Diabetes Mellitus Type 2 Patients Based on Healthentia App,” with an intraindividual control group, was approved by the General University Hospital of Larissa Scientific Committee on November 12, 2024 (protocol number 45937). The study is currently ongoing and implements the T2DM program.

## Results

### Study Overview and Demographics

The preliminary results from the “Clinical Intervention Study for Diabetes Mellitus Type 2 Patients Based on Healthentia App,” which is still ongoing with new patients being enrolled continuously, are presented in this section. The target sample size of the study is >30 patients, which has not been reached; however, the already enrolled patients provided suggestive trends and early indications. Currently, 16 patients have been enrolled. Their demographic data in terms of sex, age, and BMI are provided in Table 2. Most (11/16, 69%) were female patients aged >60 years, characterized as obese-low weight category. Out of the 16 patients, 3 (19%) almost completed the intervention (≥10th week), 6 (38%) completed the intervention, and 5 (31%) had their final clinical data captured in the system.

**Table 2:** Distribution of participants by age group, sex, and weight category (N=16).

Characteristics			Participants, n (%)
**Age group (y) and sex**			
	**31-40**		
		Male	2 (12)
		Female	1 (6)
	**41-50**		
		Male	0 (0)
		Female	1 (6)
	**51-60**		
		Male	1 (6)
		Female	2 (12)
	**61-70**		
		Male	1 (6)
		Female	6 (38)
	**71-80**		
		Male	1 (6)
		female	1 (6)
**Weight category**			
	Normal to high		3 (19)
	Overweight to low		1 (6)
	Overweight to high		3 (19)
	Obese to low		7 (44)
	Obese to high		2 (12)

### Coaching Outcomes and Statistical Analysis

The primary and secondary clinical objectives and behavioral objectives, described in the Study Design and Methods section, were assessed by 11 identified attributes. From these attributes, 6 were behavioral: weight, BMI, fasting glucose, sleep duration goal (as set by the physicians) achievement rate, steps walked, and minutes spent in moderate- or high-intensity activity. The sleep duration goal achievement was measured as the ratio of the days in a week the daily sleep duration goal was met. Intensity minutes were tracked weekly by Fitbit activity trackers and calculated as the sum of moderate-intensity minutes plus twice the number of high-intensity minutes. A total of 2 attributes were related to patient engagement, namely, the proportion of the content that patients completed (accessed to an acceptable degree) or accepted (provided positive feedback) out of the content delivered to them. The clinical objectives were assessed using 3 attributes, namely, HbA_1c_, low-density lipoprotein, and estimated glomerular filtration rate.

In the preliminary results presented in this paper, we considered the 9 patients who had (almost) completed the intervention in terms of the behavioral and engagement attributes, while only 5 patients had provided final clinical data for the clinical attributes. Patients who decided to drop out were not included in our analysis. Note that more patients are being enrolled every week; we expect to have at least 30 patients complete the study. We aim to reduce sex bias in our future enrollments. In contrast, we do not expect the bias toward higher age and BMI to be alleviated because this is the typical population afflicted by T2DM, while the geographical bias (the city of Larissa and the surrounding rural areas) will be absolute.

**Figure 6:**
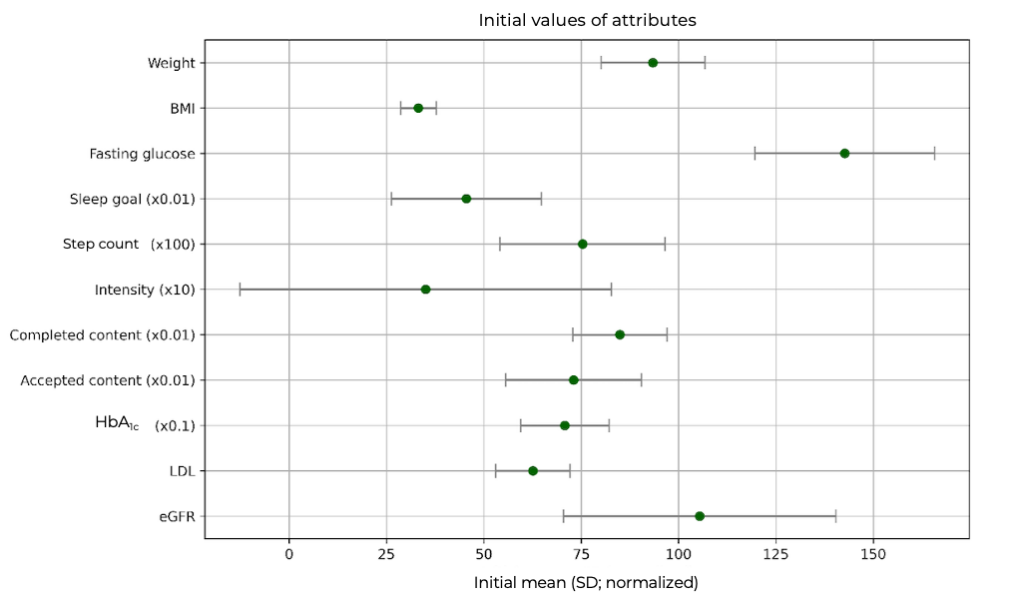
Visualization of the normalized extent of the baseline attribute values. To represent all ranges in the same graph, they are normalized in the way indicated by the multiplicative factors in the parentheses. eGFR: estimated glomerular filtration rate; HbA1c: glycated hemoglobin; LDL: low-density lipoprotein.

To evaluate the evolution of key clinical, behavioral, and engagement-related attributes over the course of the intervention, we analyzed both the initial values and their variations between baseline (assessment) and intervention completion. Table 3 summarizes the statistics (mean and SD) of the baseline attribute values. The extent of the baseline attribute values is visualized in Figure 6.

**Table 3:** Baseline characteristics of measured attributes.

Attribute	Initial values, mean (SD)
Weight (kg)	93.4 (13.3)
BMI (kg/m^2^)	33.2 (4.57)
Fasting glucose (mg/dL)	143 (23.1)
Sleep goal achievement rate	0.455 (0.192)
Steps, n	7530 (2120)
Intensity (min)	350 (477)
Completed content ratio	0.849 (0.121)
Accepted content ratio	0.730 (0.174)
HbA_1c_^a^ (%)	7.08 (1.13)
LDL^b^ (mg/dL)	62.6 (9.53)
eGFR^c^ (mL/min/1.73 m²)	105 (35)

^a^HbA_1c_: glycated hemoglobin.

^b^LDL: low-density lipoprotein.

^c^eGFR: estimated glomerular filtration rate.

Table 4 summarizes the variations observed in the attributes during the intervention, providing their mean, SDs, and relevant inferential statistics. Given the relatively small sample size in this study, sole reliance on *P* values could be misleading. While *P* values indicated whether observed differences were statistically unlikely under a null hypothesis, they were highly sensitive to sample size and might fail to capture clinically meaningful effects in underpowered studies. To mitigate this, we also reported Cohen *d*, which quantified the standardized magnitude of change regardless of sample size, and 95% CI, which offered a range of plausible values for the mean difference.

**Table 4:** Postcoaching variation and effect size.

Attribute	Variation, mean (SD)	Cohen *d*	Variation (95% CI)	*P* value
Weight (kg)	–2.89 (2.75)	1.05	(–5.00 to –0.777)	.01
BMI (kg/m^2^)	–1.05 (0.986)	1.06	(–1.80 to –0.288)	.01
Fasting glucose (mg/dL)	–17.3 (11.5)	1.50	(–26.1 to –8.43)	.002
Sleep goal achievement rate	–0.0106 (0.266)	0.0398	(–0.215 to 0.194)	.91
Steps, n	1890 (2560)	0.737	(–80.7 to 3860)	.06
Intensity (min)	150 (405)	0.371	(–161 to 461)	.30
Completed content ratio	–0.0998 (0.303)	0.329	(–0.333 to 0.133)	.35
Accepted content ratio	–0.125 (0.279)	0.447	(–0.339 to 0.0896)	.22
HbA_1c_^a^ (%)	–0.86 (1.26)	0.682	(–2.42 to 0.705)	.20
LDL^b^ (mg/dL)	1.15 (7.81)	0.147	(–11.3 to 13.6)	.79
eGFR^c^ (mL/min/1.73 m²)	–1.6 (9.91)	0.161	(–13.9 to 10.7)	.74

^a^HbA_1c_: glycated hemoglobin.

^b^LDL: low-density lipoprotein.

^c^eGFR: estimated glomerular filtration rate.

**Figure 7:**
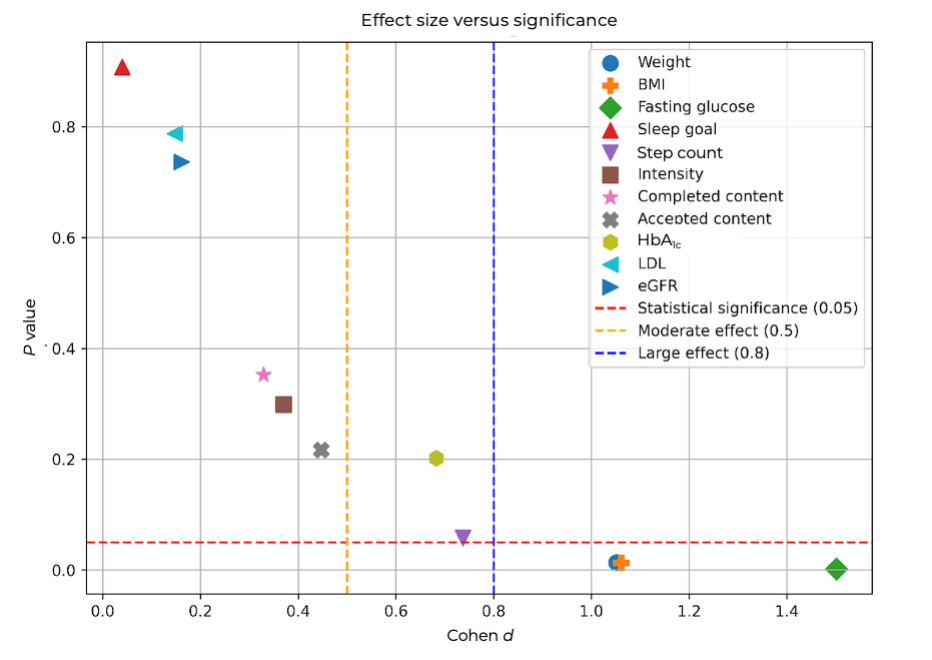
Effect size (Cohen d) versus statistical significance (*P* value) of all attributes. The thresholds for statistical significance (red horizontal dashed line), moderate effect (yellow vertical dashed line), and high effect (blue vertical dashed line) are also shown.

These complementary metrics provide more nuanced insight into intervention effects. For instance, several attributes (eg, fasting glucose and BMI) demonstrated large effect sizes and narrow CIs even when the corresponding *P* values approached conventional significance thresholds. Thus, clinical interpretation was grounded not only in statistical significance but also in the magnitude and precision of observed effects. The effect size is plotted against the statistical significance in Figure 7.

**Figure 8:**
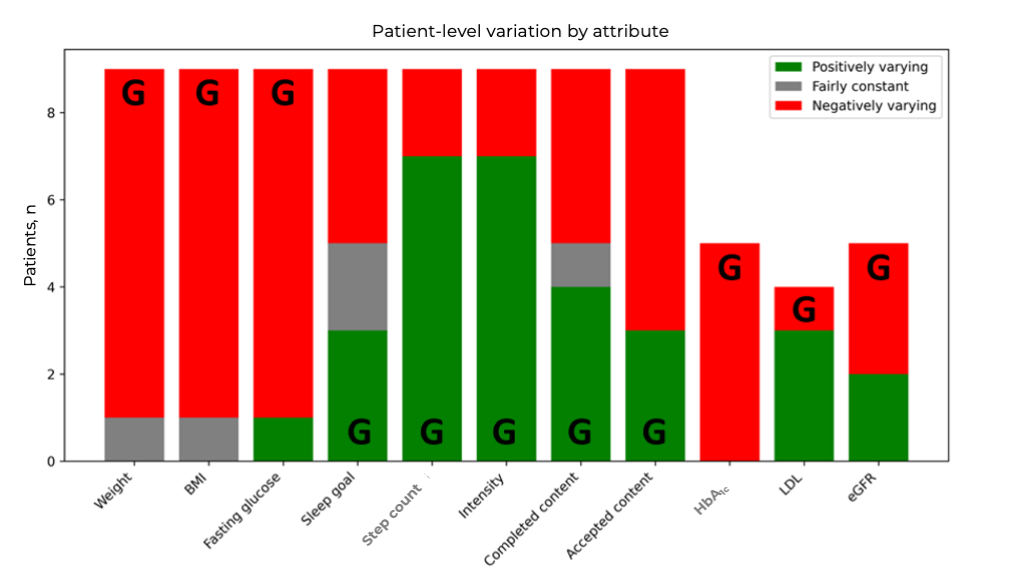
Individual patient trajectories based on relative variation for each attribute. The positive or negative variation can be a good result depending on the attribute. The good result bar is indicated with the letter “G.” For most of the attributes, more patients exhibit a good variation. eGFR: estimated glomerular filtration rate; HbA1c: glycated hemoglobin; LDL: low-density lipoprotein.

In addition to numerical trends, we categorized individual trajectories based on relative variation: patients with changes >0.5% were considered positively varying, those with changes <–0.5% as negatively varying, and the rest as fairly constant. The result is shown in Figure 8. For instance, 8 (89%) of 9 patients showed decreased fasting glucose, while sleep goal adherence was more evenly split across categories, reflecting variable behavioral compliance.

We anticipate favorable outcomes, driven at least in part by changes in patient behavior resulting from their engagement with the delivered content (refer to Table 1 for details). We consider both the volume of delivered content and its acceptance by them.

The patients could access or ignore the content items delivered to them and further provide feedback on them. Of the 1570 content items delivered thus far, 1310 (83.4%) had been accessed by the patients, and 1125 (71.7%) received positive feedback. These data are provided in Table 5 for the 3 content types and their domains.

**Table 5:** Content delivered (n=1510 content items), completed (n=1310), and positively received (n=1125) across different domains and types, showing approximate values.

Content type and domain			Delivered content items, n (%)		Completed content items, n (%)		Positively received content items, n (%)
**Tips**							
	Nutrition	150 (9.55)		150 (11.45)		150 (13.33)	
	Physical activity	20 (1.27)		10 (0.76)		10 (0.89)	
	Self-care	200 (12.74)		150 (11.45)		150 (13.33)	
	Sleep	310 (19.75)		270 (20.61)		260 (23.11)	
**Dialogues**							
	Physical activity	320 (20.38)		250 (19.08)		180 (16.00)	
	Self-care	330 (21.02)		260 (19.85)		190 (16.89)	
	Sleep	130 (8.28)		120 (9.16)		100 (8.89)	
**Lessons**							
	Self-care	110 (7.01)		100 (7.63)		85 (7.56)	

### Tailored Coaching Approaches

To illustrate the tailored nature of the BCC system, we present 2 patient cases with different behavioral domain focus areas, demonstrating how content delivery was personalized to address specific needs.

In the first case study, 2U1J0 is a female patient aged 70 years, with a baseline HbA_1c_ of 6.6% and weight of 76 kg, that required a self-care–focused coaching. On the basis of the initial COM-B assessment results, she showed low capability and motivation scores for self-care behaviors.

The BCC system delivered a higher proportion of self-care–focused content to patient 2U1J0, the first case study, compared to the overall study population average:

- The patient received 45 (57%) interactive dialogues on self-care goals out of the 79 interactive dialogues overall, compared to a study-wide average of 33 (42%).
- The patient demonstrated high engagement with the content delivered, with a 92.9% (156/168) completion rate.

Patient 2U1J0 showed improvements in self-management behaviors and clinical outcomes including a +17% increase in the Diabetes Management Self-Efficacy Scale score, satisfying weight loss of 2 kg, HbA_1c_ reduction of –0.1%, and fasting glucose reduction of 18 mg/dL.

In the second case study, FGSQN is a female patient aged 66 years, with a baseline HbA_1c_ of 8.9% and a weight of 96 kg, who reported significant barriers to physical activity. Initial assessment revealed only 5318 average daily steps.

The BCC system for patient FGSQN prioritized physical activity content:

- The patient received 42 (50%) interactive dialogues on physical activity–related goals out of the 84 interactive dialogues overall compared to a study-wide average of 35 (42%).
- The patient demonstrated high engagement with the content delivered with 89% (150/168) completion rate.

Patient FGSQN showed improvements in self-management behaviors and clinical outcomes, including an increase of daily steps by 234 (4.4% increase) counts, modest weight reduction of 1 kg, impressive HbA_1c_ reduction by 2.1%, and fasting glucose reduction of 22 mg/dL.

## Discussion

### Principal Findings

The preliminary results from the ongoing T2DM study show potential, despite being at an early stage of the study with few patients having completed the intervention phase. Three attributes fall in the statistically significant and high-effect portion of the plane of Figure 7, with favorable variations:

1. Fasting glucose levels showed the largest relative change, dropping by an average of –17.3 mg/dL (Cohend =1.5; *P*=.002), further underscored by a narrow 95% CI –26.1 to –8.43.
2. Weight and BMI, with mean reductions of –2.89 kg and –1.05 kg/m², respectively. These changes yielded large effect sizes (Cohen d approximately 1.05) and were statistically significant (*P*=.01).

Two more attributes have favorable variations with moderate effects while not statistically significant:

1. Change in steps walked has a moderate effect (Cohen d=0.737) and was close to statistical significance (*P*=.06).
2. Change in HbA1c level has a moderate effect (Cohen d=0.682), albeit far from statistical significance (*P*=.20).

For the rest of the attributes, the variation has neither at least moderate effect nor statistical significance:

- Intensity minutes attribute has a large favorable average variation. Notably, it does not fall close to the top-left portion of the effect-significance plane of Figure 7.
- Sleep goal, low-density lipoprotein levels, and estimated glomerular filtration rate levels have nonfavorable variations. Notably, they constitute the top-left portion of the effect-significance plane of Figure 7.
- Completed and accepted content both dropped during the coaching phase. This is expected because patients can be fatigued by repeated content. We plan to address this by introducing more variations to the content per domain and intervention function.

The tailored nature of this framework is presented by analyzing 2 individual cases and their delivered content that can demonstrate different coaching experiences as they have different profiles. The first patient case is focused on improving self-care behaviors, while the second patient case is focused on physical activity–related behaviors.

We next consider the content delivered to the patients. Referring to Table 5, we note a tendency to select many more tips in the sleep domain and fewer tips in the physical activity domain compared to the other 2 domains; however, the predominance of sleep-related tips declined as the study progressed. All tips are usually accessed and well received by the patients, as are the weekly lessons. The interactive dialogues on goals show a decrease in acceptance. This is not pronounced for the sleep domain, where the least number of dialogues was delivered (90/457, 19.7%), but it is for the coping and the physical activity domains, where the delivered dialogues are almost doubled (170/457, 37.2% and 197/457, 43.1%, respectively).

The poor results in terms of physical activity variation (see seventh and eighth rows of Table 3) and the acceptance of the related goal dialogues (ie, lower than the average 85%) are noted. If this trend continues, we should consider a more even pacing of the interactive dialogues on goals, even when the goals are not met.

### Limitations

The main limitation of the reported results is the limited number of patients enrolled and those who have already completed the study. This is being alleviated as the study progresses.

A second limitation stems from the inherent selection bias commonly associated with this type of study. Patients who can be convinced to participate in and complete such a study are those predisposed to cooperate, adhere to instructions, and invest the necessary time to implement the behavioral changes as instructed. This, apart from the evident role it plays in the consistency of the study population, also affects the outcome of digital coaching in various ways. Most importantly, the participants who comply are usually the same patients who already display better disease control and have less room to express improvement (ie, already have HbA_1c_ or BMI close to target values). This fact could potentially limit the strength of the study results. For example, the population of this study consists predominately of female participants, pensioners, and former civil servants.

In addition to that, the encouraging initial results stemmed from a combination of digital coaching and other factors. These combinations include the following:

- The novelty effect arises from patients participating in a care process that differs from what they have previously experienced. The coaching content delivered is the cornerstone of this novelty. The activity trackers and mobile app conclude this novelty for the participants.
- The patients are on the more digitally literate side of the spectrum. This can be an indication of higher literacy in general. It is known that higher literacy can help in the self-management of chronic diseases. It can help in ingesting the coaching content delivered.
- The patients are affected by the feedback. This includes not only the recommendations but also the information on how they are doing, presented in the form of the plots in the different app widgets.

Overall, coaching content delivered plays a role in all 3 factors. Certainly, the exact extent of the improvement that can be attributed to the recommendations cannot be fully evaluated without the use of a control group.

A final limitation of the study is the patient-reported nature of the data collection process, which leaves room for data validity to be questioned, although the patients have no incentive to input false data. Nonetheless, the characteristics of the study population are also expected to change as the study matures, and steps could be taken to mitigate such bias.

### Comparison With Prior Work

We anticipate that our ongoing study will eventually achieve better results than the study used as a reference [24], available under the German Clinical Trials Registry (DRKS00027392), which reported an average HbA_1c_ reduction of –0.9% (SD 1.1%; *P*<.001), body weight reduction of –4.3 (SD 4.5) kg, BMI reduction of –1.4 (SD 1.5) kg/m^2^, and fasting glucose reduction of –10.8 (SD 23.4) mg/dL. This expectation stems from having established the technical equivalence of Healthentia to several other applications in the study by Kyriazakos et al [25]. In addition, in contrast to other studies, we are modeling the patients’ behavior based on established theoretical models, such as COM-B, and using widely studied BCTs as methods for changing targeted behaviors tailored to individual needs. The studies using those mobile apps exhibited improvements in HbA_1c_ level, weight and BMI, and sleep duration. When the study matures, we expect improvement in all the reported attributes and the HbA_1c_ levels, and we can then safely compare the results of the reference study with the T2DM initiative.

### Conclusions

This paper introduces an end-to-end solution that integrates science-backed, personalized digital coaching with remote patient monitoring to address the challenges of chronic disease management. By combining behavioral science with digital health technologies, the approach offers a structured intervention that could drive sustainable lifestyle changes. The implementation of our framework as a part of Healthentia’s lifestyle behavioral change and wellbeing (non-medical modules) demonstrates how theoretical models can be used in practical digital health applications.

Our initial implementation of a T2DM study provides insights into the applicability and feasibility of the approach while also showing some promising but very preliminary results on the objectives, both clinical and behavioral. These include a reduction in BMI, a decrease in fasting glucose values, and a moderate reduction in HbA_1c_ levels for all patients. There is also a high engagement rate with personalized content, suggesting successful content selection.

As the T2DM program evolves and more patients complete the full program, we expect a clearer understanding of its short-term and long-term effectiveness, particularly regarding critical biomarkers, such as HbA_1c_. These insights will allow us to further refine the methodology explored and validate the impact of the approach across various patient populations and contexts.

The potential impact of this framework extends beyond individual health outcomes, promising significant reductions in health care costs through fewer hospitalizations and complications. In addition, decentralized care delivery can help mitigate the broader societal and economic burden associated with chronic disease management. Future research will explore applications to other chronic conditions and optimization of digital BCC.

## Appendix

Multimedia Appendix 1 Behavior change technique glossary and reference table.

Multimedia Appendix 2 Capability, opportunity, motivation, and behavior model questionnaires.

## Abbreviations

BCC: behavior change coaching
BCT: behavior change technique
BCW: Behaviour Change Wheel
BDI: belief-desire-intention
COM-B: capability, opportunity, motivation, and behavior
HbA1c: glycated hemoglobin
T2DM: type 2 diabetes mellitus

## Data Availability

The datasets generated or analyzed during this study are available from the corresponding author on reasonable request.
