# Supplementary material for: Multidomain Behavioral Change Digital Coaching for Chronic Disease Management in Patients With Type 2 Diabetes: Framework Development and Preliminary Evaluation
Source: JMIR Form Res. 2025 Jul 9;9:e73807. doi: 10.2196/73807 (PMC12287672; doi:10.2196/73807)
Supplement: Multimedia Appendix 1 [file formative_v9i1e73807_app1.docx]

## *Multimedia a*ppendix 1: Behavior Change Technique (BCT) Glossary and Reference Table

Table 6. This glossary provides definitions and examples of the key BCTs used in the virtual coaching system.

| Code | Name | Definition | Example of implementation in virtual coaching | Mode of delivery |
| --- | --- | --- | --- | --- |
| 1 | **Goals & Planning** | | | |
| 1.1 | Goal setting (behavior) | Set or agree on a goal defined in terms of the behavior to be achieved. | After an assessment the system tailors an intervention for the participant for the long-term goals for Steps, intensity minutes and Sleep. Short term goals are set and communicated to everyone for the upcoming week. | Dialogue |
| 1.2 | Problem solving | Analyze, or prompt the person to analyze, factors influencing the behavior and generate or select strategies that include overcoming barriers and/or increasing facilitators | The coach discusses with the participant to identify triggers or barriers that could restrict them from achieving their goal | Dialogue |
| 1.3 | Goal setting (outcome) | Set or agree on a goal defined in terms of a **positive outcome of wanted behavior**. | After an assessment the system tailors an intervention for the participant for a long-term outcome goal for weight as a result of behavioral intervention on better eating habits and more physical activity. Short term goal is communicated to each individual for the upcoming week. | Dialogue |
| 1.4 | Action planning | Prompt detailed planning of performance of the behavior | Once a goal is set or set, the coach can discuss an action plan of activities to support the individual to achieve their goals. | Dialogue |
| 1.5 | Review behavior goal(s) | Review behavior goal(s) jointly with the person and consider modifying goal(s) in light of achievement. | Daily feedback messages on goal achievement and visual displays point out the differences in set and achieved goals. Weekly the coach reviews last week's goals that may lead to a change in the goal setting, or no change. | Dialogue |
| 1.6 | Discrepancy between current behavior and goal | Draw attention to discrepancies between a person’s current behavior and goal | The system calculates the discrepancy between current and set goal. Home screen and widgets also visually display the differences between the daily set goal and the achieved progress. | System |
| 1.7 | Review Outcome goals | Review outcome goal(s) jointly with the person and consider modifying goal(s) in light of achievement. | At the end of the week, the coach starts a dialogue to review the outcome goal that may lead to a new setting of an outcome goal or may lead to review barriers 1.2 or generating new strategies 1.2 | Dialogue |
| 2 | **Feedback & Monitoring** | | | |
| 2.1 | Monitoring of behavior by others without feedback | Observe or record behavior with the person’s knowledge as part of a behaviour change strategy | This operation is done by the system in the background collecting data without necessarily giving feedback | System |
| 2.2 | Feedback on behavior | Monitor and provide informative or evaluative feedback on performance of the behavior | The coach after weekly monitoring of behavior evaluates progress and gives feedback through a dialogue | Dialogue |
| 2.3 | Self-monitoring of behavior | Establish a method for the person to monitor and record their behavior(s) | The system allows integration with a tracker that automatically collects physical activity, sleep, heart rate measurements. There are also specialized widgets that allow and prompt the participant to log | APP |
| 2.4 | Self-monitoring of outcome(s) of behavior | Establish a method for the person to monitor and record the outcome(s) of their behavior | App widget visuals allows the participant to monitor progress on weight as an outcome | APP |
| 2.6 | Biofeedback | Provide feedback about the body (e.g. physiological or biochemical state) using an external monitoring device as part of a behavior change strategy. | Daily feedback messages related to heart rate, Blood Glucose and Blood Pressure measurements | Dialogue |
| 2.7 | Feedback on outcome(s) of behavior | Monitor and provide feedback on the outcome of performance of the behavior. | The coach gives feedback upon weight measurement as the outcome of behaviour | Dialogue |
| 3 | **Social Support** | | | |
| 3.1 | Social support (unspecified) | Advise on, arrange or provide social support (e.g. from friends, relatives, colleagues,’ buddies’ or staff) or noncontingent praise or reward for performance of the behavior. It includes encouragement and counseling, but only when it is directed at the behavior. | Coach considered as a buddy/staff messages when meeting goals or when there was a good progress or gives tips on how to include friends and family in the performance of the behavior | Tips |
| 4 | **Shaping Knowledge** | | | |
| 4.1 | Instruction on how to perform a behavior | Advise or agree on how to perform the behavior (includes ‘Skills training’). | The coach prompts the participant with a recommendation (tip) or a lesson with instructions on how to perform the targeted behavior. How to eat healthy, How to shop healthier food… | Lessons, Tips |
| 4.2 | Information on antecedents | Provide information about antecedents (e.g. social and environmental situations and events, emotions, cognitions) that reliably predict performance of the behavior | The coach prompts the participant with a lesson or a course on clear factual, educational content with the necessary understanding that patients need before they can meaningfully engage with more direct behavior change | Lessons, Tips |
| 5 | **Natural Consequences** | | | |
| 5.1 | Information about health consequences | Provide information (e.g. written, verbal, visual) about health consequences of performing the behavior. | The coach prompts the participant with a recommendation (tip) or a lesson related to information on benefits of performing the behavior like eating healthier, doing physical activity but also risks and complications related to the disease | Lessons |
| 5.3 | Information about social and environmental consequences | Provide information (e.g. written, verbal, visual) about social and environmental consequences of performing the behavior. |  | Lessons |
| 5.6 | Information about emotional consequences | Provide information (e.g. written, verbal, visual) about emotional consequences of performing the behavior. |  | Lessons |
| 7 | **Associations** | | | |
| 7.1 | Prompts/cues | Introduce or define environmental or social stimulus with the purpose of prompting or cueing the behaviour. The prompt or cue would normally occur at the time or place of performance | Coach advises on how to use prompts/cues to help with performing the behavior | Tips |
| 8 | **Repetition & substitution** | | | |
| 8.2 | Behavior substitution | Prompt substitution of the unwanted behavior with a wanted or neutral behavior | Coach advises on alternative options to substitute the bad behavior | Tips |
| 8.3 | Habit formation | Prompt rehearsal and repetition of the behavior in the same context repeatedly so that the context elicits the behavior | System push notification encourages the desired behavior to formulate a habit | Push Notification |
| 8.7 | Graded tasks | Set easy-to-perform tasks, making them increasingly difficult, but achievable, until behavior is performed | System brakes the long-term plan to smaller achievable short-term goals | System |
| 9 | **Comparison of outcomes** | | | |
| 9.1 | Credible Source | Present verbal or visual communication from a credible source in favour of or against the behavior. | Article/videos from WHO or other sources in the favor of a targeted behavior | Lessons |
| 9.3 | Pros and cons | Advise the person to identify and compare reasons for wanting (pros) and not wanting to (cons) change the behavior | In the beginning or at a point when there is no progress the Coach can facilitate the identification of pros and cos with the individual | Dialogue |
| 10 | **Reward and threat** | | | |
| 10.3 | Non-specific reward | Arrange delivery of a reward if and only if there has been effort and/or progress in performing the behavior | A notification is sent to congratulate for achieving the daily goal | Push Notification |
| 12 | **Antecedents** | | | |
| 12.1. | Restructuring the physical environment | Change, or advise to change the physical environment in order to facilitate performance of the wanted behavior or create barriers to the unwanted behavior | Coach advises to facilitate behavior | Tips |
| 12.2 | Restructuring the social environment | Change, or advise to change the social environment in order to facilitate performance of the wanted behavior or create barriers to the unwanted behavior | Coach advises to facilitate behavior | Tips |
| 12.3 | Avoidance/reducing exposure to cues for the behavior | Advise on how to avoid exposure to specific social and contextual/physical cues for the behavior, including changing daily or weekly routines | Coach advises to facilitate behavior | Tips |
| 15 | **Self-belief** | | | |
| 15.1. | Verbal persuasion about capability | Tell the person that they can successfully perform the wanted behavior, arguing against self-doubts and asserting that they can and will succeed | Message on boosting confidence to do the daily goals of the behaviour | Tips |
| 15.3. | Focus on past success | Advise to think about or list previous successes in performing the behavior | If adherence is low but in the past there was success you can use it as | Dialogue |
