# Supplementary material for: Multidomain Behavioral Change Digital Coaching for Chronic Disease Management in Patients With Type 2 Diabetes: Framework Development and Preliminary Evaluation
Source: JMIR Form Res. 2025 Jul 9;9:e73807. doi: 10.2196/73807 (PMC12287672; doi:10.2196/73807)
Supplement: Multimedia Appendix 2 [file formative_v9i1e73807_app2.docx]

## *Multimedia a*ppendix 2: COM-B Questionnaires

All questionnaires have a 7-point scale ranging from 1= strongly disagree to 7 = strongly agree and are based on Murphy et al. [10].

Table 7. COM-B questionnaire for PA with the question: What needs to happen for your physical activity to increase?

| **COM** | | **TDF** | **COM question** |
| --- | --- | --- | --- |
| **Capability** | | | |
|  | Ph | Skills | Having better physical skills to do physical activity |
|  | Ps | Knowledge | Knowing more about physical activity and how it relates to my health |
|  | Ps | Cognitive skills | Having better skills to plan and be consistent in doing physical activity |
|  | Ps | Cognitive skills | Having better skills to monitor my physical activity |
|  | Ps | memory, processes | Developing the willpower to do physical activity |
| **Opportunity** | | | |
|  | So | Social influences | Having more support from others to do physical activity |
|  | Ph | Environmental context & resources | Having more triggers to prompt me to do physical activity |
|  | Ph | Environmental context & resources | Having more time to do physical activity |
| **Motivation** | | | |
|  | Re | Intentions and goals | Feeling more motivated to do physical activity |
|  | Re | Beliefs about consequences | Feeling confident when I do physical activity |
|  | Au | action planning | Developing a habit for doing physical activity |
|  | Au | Emotions | Having positive emotions about doing physical activity |

Table 8. COM-B questionnaire for Sleep with the question: What do you think would it take for you to have better sleeping habits?

| **COM** | | **TDF** | **COM question** |
| --- | --- | --- | --- |
| **Capability** | | | |
|  | Ph | Skills | Having better skills to fall asleep |
|  | Ps | Knowledge | Knowing more about the recommended sleep guidelines and how these relate to my health |
|  | Ps | Cognitive skills | Having better skills to plan and be consistent to a sleeping routine |
|  | Ps | Cognitive skills | Having better skills to monitor my sleep |
|  | Ps | memory, processes | Developing the willpower to follow a good sleeping routine |
| **Opportunity** | | | |
|  | So | Social influences | Having more support from others to make follow good sleeping routine |
|  | Ph | Environmental context & resources | Having more triggers to prompt me to fall asleep |
|  | Ph | Environmental context & resources | Having more time to sleep |
| **Motivation** | | | |
|  | Re | Intentions and goals | Feeling more motivated to follow a good sleeping routine |
|  | Re | Beliefs about consequences | Feeling confident about the ability to falling asleep |
|  | Au | action planning | Developing a habit of a good sleeping routine |
|  | Au | emotions | Having positive emotions about a good sleeping routine |

Table 9. COM-B questionnaire for Nutrition: What do you think it would take for you to make healthier eating habits?

| **COM** | | **TDF** | **COM question** |
| --- | --- | --- | --- |
| **Capability** | | | |
|  | Ph | Skills | Having better skills to prepare healthy meals |
|  | Ps | Knowledge | Knowing more about the recommended dietary guidelines and how these relate to my health |
|  | Ps | Cognitive skills | Having better skills to plan and be consistent in eating healthy |
|  | Ps | Cognitive skills | Having better skills to monitor my dietary intake |
|  | Ps | memory, processes | Developing the willpower to eat healthier |
| **Opportunity** | | | |
|  | So | Social influences | Having more support from others to make healthier food choices |
|  | Ph | Environmental context & resources | Having more triggers to prompt me to make healthier food choices |
|  | Ph | Environmental context & resources | Having more time to choose and prepare healthy meals |
| **Motivation** | | | |
|  | Re | Intentions and goals | Feeling more motivated to eat healtheir |
|  | Re | Beliefs about consequences | Feeling confident when I do physical activity |
|  | Au | action planning | Developing a habit for eating healthy |
|  | Au | emotions | Having positive emotions about eating healthy |

Table 10. COM-B questionnaire for self-care: What do you think it would take for you to better self-care for your condition?

| **COM** | **TDF** | **COM question** | **COM** |
| --- | --- | --- | --- |
| **Capability** | | | |
|  | Ph | Skills | Having better skills to self-care |
|  | Ps | Knowledge | Knowing more about the recommended self-care guidelines and how these relate to my health |
|  | Ps | Cognitive skills | Having better skills to plan and be consistent to self-care for my condition |
|  | Ps | Cognitive skills | Having better skills to monitor my condition |
|  | Ps | memory, processes | Developing the willpower to self-care |
| **Opportunity** | | | |
|  | So | Social influences | Having more support from others to self-care for my condition |
|  | Ph | Environmental context & resources | Having more triggers to prompt me to self-care for my condition |
|  | Ph | Environmental context & resources | Having more time to self-care for my condition |
| **Motivation** | | | |
|  | Re | Intentions and goals | Feeling more motivated to self-care for my condition |
|  | Re | Beliefs about consequences | Feeling confident when I self-care for my condition |
|  | Au | action planning | Developing a habit for self-care |
|  | Au | emotions | Having positive emotions about self-caring for my condition |
